# Deep Learning for Content-Based Medical Image Retrieval in Picture Archiving and Communication Systems for Brain Tumor Detection: Algorithm Development and Validation

**DOI:** 10.2196/78300

**Published:** 2026-04-06

**Authors:** Chin-Lin Lee, Tzu-Hsuan Hsu, Yu-Te Wu, Wan-Yuo Guo, Woei-Chyn Chu, Chung-Yueh Lien

**Affiliations:** 1 Department of Information Management, National Taipei University of Nursing and Health Science, No. 365, Ming-te Rd, Beitou Dist, Taipei City, 112303, Taiwan, +886 2-2822-7101 ext 1230; 2 Institute of Biophotonics, National Yang Ming Chiao Tung University, Taipei, Taiwan; 3 Department of Radiology, Taipei Veterans General Hospital, Taipei, Taiwan; 4 Department of Biomedical Engineering, National Yang Ming Chiao Tung University, Taipei, Taiwan

**Keywords:** content-based medical image retrieval, CBMIR, deep learning, Digital Imaging and Communications in Medicine, DICOM, brain tumor, picture archiving and communication system, PACS, tumor

## Abstract

**Background:**

Advances in medical imaging have led to massive archives, yet navigating these datasets remains challenging due to the limitations of traditional text-based search engines. While content-based medical image retrieval (CBMIR) offers a visual feature–based solution to enhance clinical workflows and research, its operational integration into picture archiving and communication systems (PACS) remains a significant bottleneck. Despite the progress in deep learning for feature extraction, CBMIR tools are rarely integrated and effectively implemented within existing radiology information systems due to complex protocol barriers.

**Objective:**

To address these challenges, this study develops a CBMIR system meticulously designed to cater to 7 distinct types of brain tumors as seen in brain magnetic resonance images. Our system is tailored to assist radiologists and health care professionals in efficiently retrieving pertinent historical medical images, thereby providing quantitative decision support for radiologists and facilitating evidence-based case comparison, with the potential to improve retrieval efficiency and clinical workflow, rather than directly enhancing diagnostic accuracy.

**Methods:**

The dataset used in this study was collected from a single medical center and is not publicly available. The core innovation is a state-of-the-art deep learning–based feature extraction algorithm specifically engineered for the CBMIR system. We use GoogLeNet as the primary architecture, incorporating generalized mean pooling to capture nuanced local features and an embedding layer for dimension reduction. Crucially, we address the integration gap by harmonizing 2 open-source projects to successfully embed the CBMIR system into a functional PACS environment via standard protocols.

**Results:**

The image dataset contains 658 participants with 15,873 images collected from 2000 to 2017. The empirical findings of our research demonstrate the performance and robustness of the proposed CBMIR system. Our system achieves a remarkable mean average precision score of 89.16% and an equally impressive Precision@10 score of 94.08%. These metrics affirm the system’s efficacy in retrieving relevant medical images. Furthermore, we successfully integrate the CBMIR system into a PACS by successfully harmonizing 2 open-source projects.

**Conclusions:**

This study presents the design and implementation of a PACS-integrated CBMIR system for brain magnetic resonance imaging, and the experimental results demonstrate that the system can achieve efficient and accurate image retrieval within a clinical workflow.

## Introduction

### Background

Advances and rapid growth in medical imaging technology have led to the emergence of massive medical image archives. Effective image retrieval is crucial for both research and clinical applications, yet navigating these large-scale archives remains a time-consuming task, often limited by traditional text-based search engines [1]. Radiologists, in particular, face the challenge of interpreting cross-sectional studies composed of thousands of images retrieved from picture archiving and communication systems (PACS) [2].

PACS is a health care IT system that manages the storage, retrieval, distribution, and presentation of medical digital images within a hospital. It works closely with the DICOM (Digital Imaging and Communications in Medicine) standard, which ensures that medical images and related data are stored and transmitted in a consistent format across different machines and systems. To integrate a third-party system, it must interface directly with the PACS via the DICOM protocol. This integration is critical as it allows health care providers to successfully access and share patient images within their workflows, enabling the content-based medical image retrieval (CBMIR) system to function effectively as a clinical decision support tool.

CBMIR offers a more efficient solution by enabling image searches based on the visual features of source images. CBMIR systems have the potential to significantly enhance clinical imaging workflows, delivering meaningful improvements across multiple areas, including medical education, research, and clinical diagnostics [3].

Röhrich et al [4] found that integrating CBMIR into medical imaging reduced image interpretation time by 31.3% for both junior and senior radiologists, while also helping them access more valuable consultative resources. When combined with an intelligent interactive visual browser, CBMIR could further enhance medical education, research, and clinical care by improving both image navigation and diagnostic outcomes [1]. Müller et al [5] highlighted the usefulness of CBMIR in academic settings, particularly for lecturers and students who can visually explore educational image repositories, facilitating more engaging and insightful learning experiences.

The retrieval principle encompasses several key aspects that collectively contribute to the effective retrieval of information. In particular, intricate processes are involved, such as analyzing and converting user requisites into queries suitable for retrieving data from indexed databases. In the process of CBMIR operation, the features of the images, relying on captions, visual words [6], preannotations [7], and image attributes [8], are first extracted and stored in a database. A distance metric summarizing information about image features measures the similarity between any given pair of images [9,10]. During CBMIR development, the key components are feature extraction and similarity determination [8], with feature extraction being the crucial step. In the past, CBMIR techniques have focused on semantic content matching of images, for example, analyzing and retrieving images based on their color, texture, and layout. Ponciano-Silva and colleagues [11] addressed the limitations of past CBMIR systems that typically used only a single perceptual parameter (ie, feature extraction and distance function), thus restricting such systems to a single search space.

In recent years, the rapid development of artificial intelligence (AI) technology and significant improvements in image processing capabilities have led to the application of AI technology to enhance CBMIR performance. The powerful algorithmic capabilities of deep learning have also been applied to CBMIR systems. Maji et al [12] compared the accuracy of various AI pretrained models, such as DenseNet [13], InceptionResNetV2 [14], InceptionV4 [14], MobileNetV2 [15], ResNet50 [16], and VGG19 (Visual Geometry Group model 19) [17]. They concluded that InceptionResNetV2 exhibited the highest accuracy and chose it as the feature extraction model. They further proposed using principal component analysis and clustering methods to increase retrieval speed by at least a factor of 2.

Rashad et al [18] adopted pretrained deep learning models to extract additional high-level features from medical images to improve the accuracy of the retrieved results in extracting compact, deep, and high-level features. Cheng and colleagues [19] tackled the limited texture representation of brain tumor images in traditional image processing methods. They used the Fisher Vector to aggregate local features into a single vector and used closed-form metric learning to effectively measure similarity between 2 given images. They achieved a mean average precision (mAP) of 94.8%. While this method achieved excellent accuracy, the concatenation of feature vectors from various subregions led to a high dimensionality of up to 24,576, potentially resulting in decreased query speed.

Qayyum et al [20] collected a diverse medical image dataset encompassing 24 anatomical regions and various imaging modalities. They used deep learning to train and build an 8-layer model whose architecture transforms 4096 features into a probability distribution over 24 categories. The average accuracy achieved in classification was 99.76%. They found that without prior classification and querying, mAP reached a mere 53%, while performing classification before querying improved mAP to 69%. Swati et al [21] focused on a contrast-enhanced magnetic resonance imaging brain tumor dataset [22] and used the pretrained VGG19 model (Visual Geometry Group model 19) for fine-tuning transfer learning (TL). Their study applied deep learning by using the last fully connected layer to extract the features, which is the input of the closed-form metric learning for contrast-enhanced magnetic resonance imaging to achieve a mAP of 96.13% [21]. Deepak and Ameer [23] evaluated various pretrained model architectures in terms of classification accuracy, training time, and feature extraction time. They selected GoogLeNet as the basis for their deep learning architecture, requiring a 2-stage training process; the achieved mAP was 97.64%.

Vision Transformer (ViT)–based models have demonstrated significant advantages when dealing with large datasets, enabling them to attend to critical image features, superior capacity for global feature modeling and context awareness, and thereby improve medical image classification performance [24]. For instance, Anupama and Anitha [25] developed a ViT-based CBMIR system that reliably and precisely extracts pertinent medical images. The predicted results, particularly for magnetic resonance imaging data, confirm the model’s capability to manage intricate imaging characteristics and provide precise outcomes [25]. Furthermore, studies by Sunday et al [26] highlight the use of the DINOv2 self-supervised framework to enhance anatomical structure visibility and reduce image noise. This demonstrates the potential of general-purpose self-supervised models in CBMIR, offering high transferability and robustness to varying image quality without requiring extensive domain adaptation [26]. In related work, Sarıateş and Özbay [27] used a TL framework to successfully classify melanoma images and enhance the efficiency of their content-based image retrieval systems.

However, due to technological bottlenecks that have yet to be overcome, CBMIR is rarely used in existing PACS and radiology information systems [1]. CBMIR could greatly benefit researchers and physicians in PACS [28-31]. Valente et al [32] used the open-source PACS software Dicoogle (University of Aveiro) as the development base for CBMIR for clinical applications. Besides general image, they focused on a mammography profile specifying the mammography features, allowing users to access CBMIR features integrated with a PACS to ensure high scalability.

### Our Contributions

Our primary contributions are summarized as follows: This study proposes an innovative CBMIR approach using ArcFace Loss [33] to enhance the understanding of image feature similarity and replaces the pooling layer with generalized mean (GeM) pooling. This allows the model to focus on key regions while preserving overall image information. This strategy strengthens the feature space’s discriminability, resulting in significantly improved retrieval accuracy (mAP). We developed a robust CBMIR system successfully integrated with open-source DICOM viewer named BlueLight [34], achieving real-time clinical applicability by enabling rapid consultation of structurally similar cases.

## Methods

### Dataset

We gathered from Taipei Veterans General Hospital (TVGH) a comprehensive collection of brain magnetic resonance (MR) images formatted in DICOM. This image dataset was retrospectively collected and features a wide array of neurological conditions. These conditions have been categorized into 7 distinct groups: metastasis, glioma, meningioma, pituitary adenoma, vestibular schwannoma, and categories for images with no tumor contour and those representing normal brain scans.

**Figure 1.**
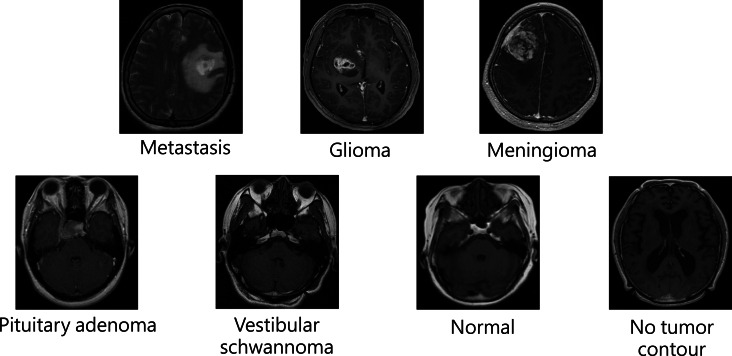
Examples of all tumor classes.

All classes of tumors were delineated by neurosurgeons and validated by neuroradiologists from the TVGH, referred to as “ground truth.” Table 1 provides statistics regarding the classes of tumors in the dataset. The image dataset, comprising 658 participants, was divided into training (70%, 461 participants, 11,207 images) and test (30%, 197 participants, 4666 images) subsets collected from 2000 to 2017. The median age was 56.4 years. Gender distribution was 57% (375/658) male and 43% (283/658) female. The distribution of image laterality was 52% left and 48% right. The median tumor volume, excluding normal images, was 2.02 mL, calculated from manual segmentation of lesions in MR images. Some patients underwent Gamma Knife radiosurgery that contains both pre–radiation therapy images and follow-up images. No patient overlapped between the training and test sets. Data in the test subset were not augmented. Examples of all classes of tumors are shown in Figure 1.

**Table 1.** Classification of tumors in the dataset.

Class	Training set	Test set	Total
Metastasis	1479	651	2130
Glioma	1484	633	2117
Meningioma	1466	632	2098
Pituitary adenoma	1520	629	2149
Vestibular schwannoma	1492	645	2137
No contour	2387	884	3271
Normal brain	1379	592	1971
Overall	11,207	4666	15,873

A brain image is inherently composed of a series of image slices, constituting a set that encompasses images both with and without tumor contours. In real-world clinical scenarios, such a dual presence of image types is a common occurrence. Focusing solely on images displaying tumors would inadvertently result in the exclusion of those lacking tumor contours, thereby impeding the accurate extraction of the latter. To mitigate this challenge, we implemented a strategy to segregate such images into a distinct category labeled “no tumor contour.” This deliberate classification enables us to effectively handle and differentiate images without tumor contours, thereby enhancing the comprehensiveness of our dataset and ensuring a more accurate analysis.

### Imaging Parameters

All images were acquired using consistent imaging acquisition protocols, detailed as follows. MR scans were obtained using 1.5T parametric MR scanners from manufacturers General Equipment and Philips. Each image had a spatial resolution of 512×512 pixels, with 16-bit depth per pixel, and a slice thickness of 3 mm. The imaging protocols included both T1-weighted (T1W) and T2-weighted (T2W) sequences. These sequences were conducted as follows: (1) T1W+C: acquired using axial 2D spin echo (repetition time = 416 ms, echo time = 9 ms, flip angle = 90°). (2) T2W: acquired using axial 2D spin echo (repetition time = 4050 ms, echo time = 109 ms, flip angle = 90°).

### Image Preprocessing and Augmentation

The original 512×512-pixel single-channel DICOM images were resized to 224×224 pixels using bilinear interpolation. Although the GoogLeNet backbone, integrated with GeM pooling, supports variable spatial dimensions, this specific scale was selected to align with the ImageNet pretraining distribution. Such standardization optimizes the balance between representation learning and computational efficiency, significantly reducing graphics processing unit memory footprint and inference latency. To leverage pretrained weights without altering the input layer, the resulting grayscale images were stacked into 3 identical channels (red-green-blue). Finally, while the training set underwent data augmentation (including random rotation, flipping, and scaling) to enhance robustness, the test set remained nonaugmented to ensure an evaluation reflective of clinical practice. This choice represents a practical trade-off between computational efficiency and spatial resolution, which is appropriate for large-scale similarity retrieval rather than pixel-level diagnosis.

### System Architecture

**Figure 2.**
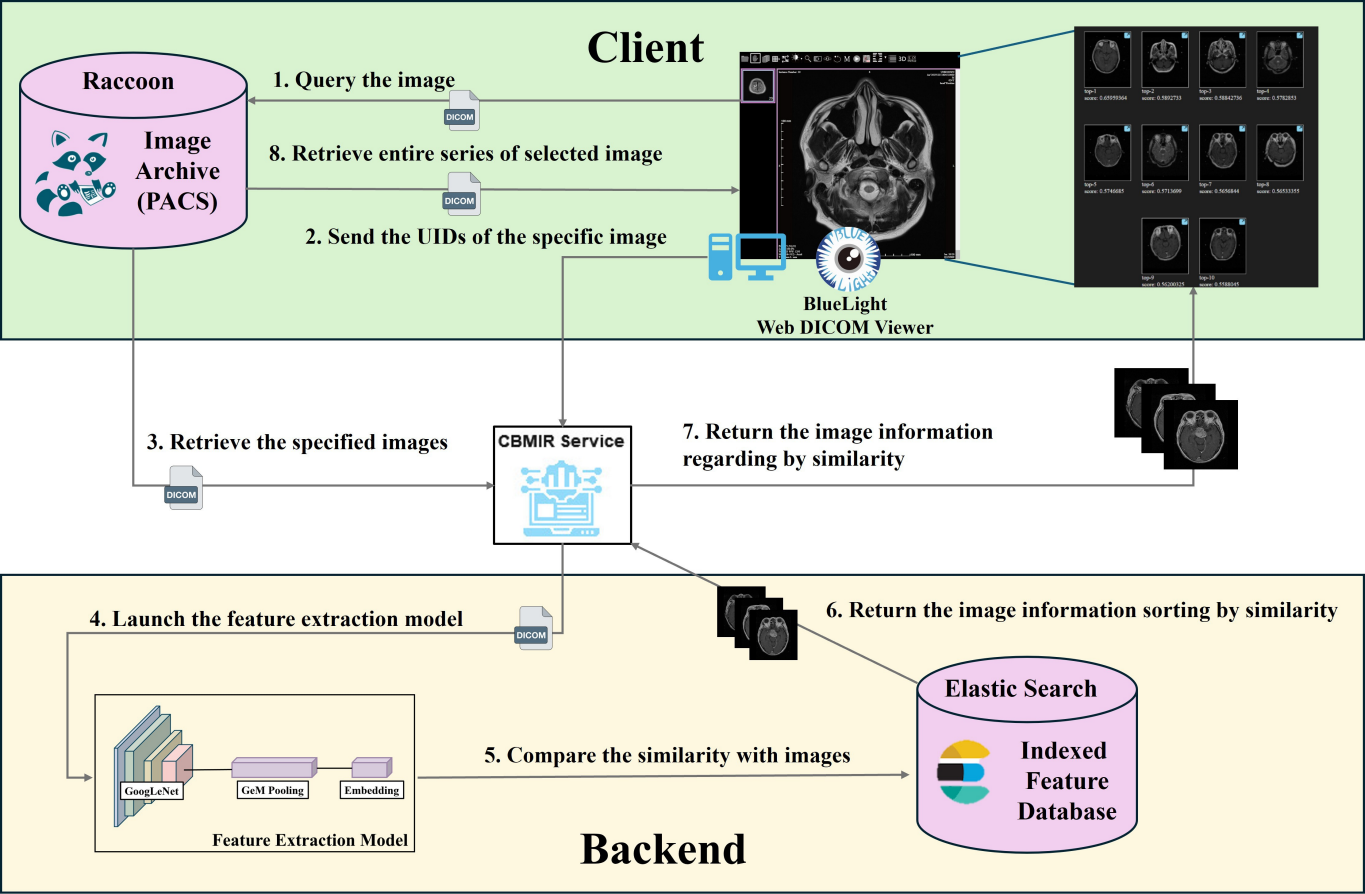
Feature extraction model and content-based medical image retrieval service architecture. CBMIR: content-based medical image retrieval; DICOM: Digital Imaging and Communications in Medicine; PACS: picture archiving and communication systems; UIDs: unique identifiers.

The CBMIR system, including the feature extraction model, is illustrated in Figure 2. As seen therein, radiologists can select the images they wish to query for the CBMIR system within the web DICOM viewer. Upon selection, the query image is initially input into a pretrained AI model for feature extraction. Subsequently, CBMIR takes these feature vectors and matches them against the database, retrieving images with similar feature vectors from the database. For each search, the user sends a single query request containing 3 DICOM UIDs: the Study Instance UID, Series Instance UID, and SOP Instance UID. In response, the CBMIR system retrieves the specified image from the PACS and sends it to the feature extraction model. After processing, the retrieved images are then presented in the web DICOM viewer. Finally, the user can select the most closely related image and retrieve the entire study from the PACS server.

### Feature Extraction Model

The foundational network of the feature extraction model architecture is based on GoogLeNet, with a modification replacing the original global average pooling with GeM pooling [35] to obtain more generalized features. Finally, an embedding layer is added to reduce the feature dimensions from 1024 to 256, compressing the data for improved computational efficiency. An explanation of each of the components of the model follows in the subsections, including (1) GoogLeNet, (2) GeM pooling, (3) embedding, and (4) the loss function.

1. *GoogLeNet layer*: The primary design core is the inception architecture, which uses 1×1 convolutional layer to reduce dimensionality, thereby lowering computational complexity. The inception architecture also splits the input into various sizes for parallel processing and subsequently aggregates them to extract features at various scales. The choice of GoogLeNet as the network backbone in this study is influenced by a previous study [23]. According to their results, GoogLeNet demonstrates several advantages over other potential network backbones, including lower computational complexity (number of operations), minimal memory consumption (parameter count), higher validation accuracy, shorter training time, and faster feature extraction. Furthermore, although ResNet50 exhibits higher accuracy, its training time is approximately 3 times that of GoogLeNet. Therefore, ResNet50 was not considered for this study due to our preference for efficient computation.
2. *GeM pooling layer*: GeM pooling was proposed by Ponciano-Silva et al [11] as a method that uses a universal formula to replace traditional global max pooling, that is,

  (1) $$f^{\left(m\right)}={\left[f_{1}^{\left(m\right)}\ldots f_{k}^{\left(m\right)}\ldots f_{K}^{\left(m\right)}\right]}^{⊤},f_{k}^{\left(m\right)}=\underset{x\in X_{k}}{max}x,$$

and global average pooling, that is,


(2) $$f^{\left(a\right)}={\left[f_{1}^{\left(a\right)}\ldots f_{k}^{\left(a\right)}\ldots f_{K}^{\left(a\right)}\right]}^{⊤},f_{k}^{\left(a\right)}=\frac{1}{\left|X_{k}\right|}\sum_{x\in X_{k}}x$$

Formula equation (3) shows the process of GeM pooling, which uses a 3D tensor *x* with dimensions *W* × *H* × *K* as input to compute the result, represented as the *f* vector.

   (3) $$f^{\left(g\right)}={\left[f_{1}^{\left(g\right)}\ldots f_{k}^{\left(g\right)}\ldots f_{K}^{\left(g\right)}\right]}^{⊤},f_{k}^{\left(g\right)}={\left(\frac{1}{\left|X_{k}\right|}\sum_{x\in X_{k}}x^{p_{k}}\right)}^{\frac{1}{p_{k}}}$$

In this formula, *pk* is a parameter that determines the norm level. When *pk*→∞, GeM pooling becomes equivalent to max pooling; when *pk*=1, it simplifies to average pooling. Although *pk* can be treated as a trainable parameter, in this study, it was fixed to *pk* = 3 for all experiments, following prior image retrieval benchmarks [35]. This design choice ensures a stable and reproducible feature embedding space across all evaluations.

By maintaining *pk* as a constant rather than a trainable parameter, we ensure a stable feature embedding space, particularly when processing domain-specific medical images. This approach contributes to improved accuracy and consistency in image retrieval tasks.

3. *Embedding layer*: The embedding layer exists primarily to reduce the dimensionality of the output from the global pooling layer. In this study, we used an embedding structure [33,36] that consists of a sequence of FC (fully connected) − BN (BatchNorm) − PReLU (parametric rectified linear unit) layers. The FC layer maps the original 1024-dimensional features to 256 dimensions while attempting to preserve the original features as much as possible. The BN layer normalizes the values to mitigate the problem of vanishing gradients, and the PReLU layer adds nonlinearity to the model.
4. *Loss function*: This study uses the ArcFace loss function [33], as represented in formula equation 4.

   (4) $$L=\frac{-1}{b}log\frac{e^{s\left(cos\left(\theta_{y_{i}}+m\right)\right)}}{e^{s\left(cos\left(\theta_{y_{i}}+m\right)\right)}+\sum_{j=1,j\neq y_{i}}^{n}e^{scos\theta_{j}}}$$

In this formula, *b* represents the batch size, *s* corresponds to the fixed feature values after L2-normalization,$\theta_{y_{i}}$ represents the weight and angle between the correct class and feature, and *m* controls the additional angle for separating different classes. The primary objective of this loss function is to ensure that embedding features from different classes are pushed as far apart as possible while keeping embedding features from the same class close together.

### Enhanced Feature Discriminability and Aggregation Strategy

In our feature extraction model, 2 key techniques were used to optimize the quality and representativeness of the feature vectors.

ArcFace loss significantly increases the quality and purity of the feature clusters. This robust separation ensures that when the system queries an image, the retrieved results are demonstrably and reliably from the most similar cluster, thus leading to higher retrieval accuracy and confidence.

GeM Pooling allows the network to intelligently decide where to focus its attention for feature extraction. It enables the model to effectively highlight the most relevant regional features (like specific pathological textures) while retaining sufficient global structural context for comprehensive retrieval, making the resulting feature vector more representative of the entire image.

### Indexed Features

We use Elasticsearch as the indexing database due to its native support for high-dimensional vector storage that supports a data type for feature vectors and can perform similarity evaluations on vectors that enable retrieval of data results sorted by similarity. The features are 1024-dimensional embeddings extracted from the GoogLeNet backbone. To enhance searching performance, the indexing phase involves the following steps:

For the core similarity calculation between feature vectors (per image), we adopt the Euclidean Distance defined in formula equation 5. This metric calculates the straight-line geometric distance between 2 feature vectors in the high-dimensional space. The retrieval results are then precisely ranked based on this distance, where a smaller distance signifies higher similarity.

To significantly enhance retrieval efficiency, we used a precalculation strategy. During the indexing phase, we precalculate and store the similarity difference values between each image and all other images in the database. Each image record is uniquely identified by 3 UIDs: Study instance UID, Series instance UID, and SOP instance UID. When a new image is queried, the system calculates its distance to the query and uses this preestablished similarity difference lookup table for comparison, which avoids the traditional method of sequentially searching and calculating every single entry.

It should be noted that this precalculation strategy is primarily designed for intradatabase browsing and retrospective analysis and applies only to images that have already been indexed. For new query images not included in the database, similarity computation is performed on the fly using the Hierarchical Navigable Small World (HNSW) algorithm, which is the default vector search method provided by Elasticsearch. Specifically, when a new query image is submitted, its feature vector is extracted and compared against the indexed feature vectors using HNSW-based approximate nearest neighbor search.

### Similarity Calculation

The similarity between feature vectors of different samples is typically measured using distance metrics to gauge the closeness or dissimilarity of each sample. In this study, we used the Euclidean distance, as represented in formula equation 5.

 (5) $$\begin{array}{l} D\left(p,q\right)=\sqrt{{\left(p_{1}-q_{1}\right)}^{2}+{\left(p_{2}-q_{2}\right)}^{2}+\ldots +{\left(p_{n}-q_{n}\right)}^{2}}\\ Similarity\;sore\left(p,q\right)=\frac{1}{D\left(p,q\right)} \end{array}$$

Here, *p* represents the feature vector of the image that the user wishes to query, and *q* represents the feature vector of the image stored in the database. The Euclidean distance measures the real distance between any 2 elements in an *n*-dimensional space. While a smaller Euclidean distance mathematically signifies higher feature-level similarity, we transformed these raw distances into “Similarity Scores” via an inverse distance mapping for display within the CBMIR interface. This ensures that higher scores directly reflect closer visual matches, thereby aligning the computational output with standard clinical assessment logic. This design choice aligns with the study’s objectives by leveraging the capacity of deep learning models to encode semantic relationships as geometric distances within a high-dimensional feature space, while ensuring that the retrieval outputs are presented in a format intuitive for clinical practitioners.

### Evaluation Factors

Once the CBMIR system is developed, objective evaluation metrics are essential to assess its performance. In this context, mAP and Precision@10 are commonly used evaluation measures [19,21,23]. mAP is extended from precision and average precision (AP). Precision is a fundamental metric and is defined as

  (6) $$Precision=\frac{Relevant\;images\cap Retrieved\;images}{Retrieved\;images}.$$

Precision is often computed as Precision@k for various values of *k*, where *k* is typically set to 10, 20, or 30. This computation helps determine the performance of the retrieval results when a certain number of images are returned. However, precision provides insight into performance only at a specific rank. AP extends precision by considering all ranks of retrieved images. AP is defined as


(7) $$AP=\frac{\sum_{k=1}^{n}Precision\left(k\right)\times rel\left(k\right)}{Relevant\;images},\;rel(k)=\left\{ \begin{array}{l} 1,\wedge if\;image\;at\;k\;is\;relevant\;image\\ 0,\wedge otherwise. \end{array}\right.$$

Here, *rel*(*k*) is a binary indicator, assuming a value of 1 if the image at rank *k* is relevant and 0 otherwise. AP provides a more comprehensive view of retrieval performance than ordinary precision. However, AP is typically used for a single query; to assess retrieval performance across multiple queries, mAP is used. mAP is defined as

  (8) $$mAP=\frac{\sum_{q=1}^{n}AP\left(q\right)}{Q}.$$

Here, *Q* represents the total number of queries, and *q* denotes the current query number. mAP gives an aggregate performance measure across multiple queries, providing a more comprehensive evaluation of the CBMIR system’s effectiveness than ordinary precision measures could provide.

### Ethical Considerations

This study was conducted according to the guidelines of the Declaration of Helsinki and approved by the Institutional Review Board (1) of TVGH (protocol code 021-05-0005CC; approval date: March 14, 2023). Informed consent was waived because of the retrospective nature of the study and the use of deidentified clinical data.

## Results

### Overview

In this study, the image dataset contains 658 participants with 15,873 images collected from 2000 to 2017. The experimental environment for this study used the following hardware specifications: an AMD Ryzen 3 PRO 4200G CPU, 24 GB of DDR4 memory, and a dedicated Nvidia GeForce RTX 3060 Ti Graphics Card. The graphics processing unit features 4864 CUDA cores and 8 GB of GDDR6 video memory. The operating system used was Linux Ubuntu (version 22.04; Canonical). For software development, the primary Integrated Development Environment used was PyCharm (JetBrains). The programming language was Python (Python Software Foundation), and the deep learning framework used for code development was PyTorch (PyTorch Foundation).

### Evaluation of AI Models for Feature Extraction Model

In evaluating the base feature extraction model for the CBMIR system, our core focus was centered on balancing model performance with clinical use and efficiency. To ensure that the system delivers fast, effective retrieval services within a real-world clinical workflow, we conducted a comprehensive comparative analysis of several state-of-the-art AI models when evaluated on the dataset. This comparison was intended to assess how modern, large-scale ViT architectures perform on the dataset relative to established CNNs (Convolutional Neural Networks).

For consistency and to leverage learned feature representations, TL was applied for all models. Each model was initialized with ImageNet pretrained weights and subsequently fine-tuned on our dataset. To explicitly examine the trade-off between model capacity and dataset size, we evaluated both base-scale ViT architectures (Swin-Base and DinoV2-Base) and their lightweight variants (Swin-Tiny and DinoV2-Small). The comparative results incorporating 95% CIs are shown in Table 2.

The results indicate that, under this TL setting and dataset scale, ViT-based models exhibit higher computational complexity and do not achieve superior retrieval performance compared with the CNN-based approach. In contrast, GoogLeNet demonstrates consistent and robust superiority across accuracy, mAP, *F*_1_-score, and computational efficiency. As evidenced by the clearly separated CIs in Table 2, GoogLeNet (95.9% accuracy) substantially outperforms the second-best architectures (upper bound 87.1%), supporting that its performance advantage is stable and unlikely to result from sampling variability. Therefore, within the context of TL on a specific clinical magnetic resonance imaging dataset, GoogLeNet was selected as the backbone feature extractor for the proposed CBMIR system.

**Table 2.** Comparative performance and efficiency of different artificial intelligence models for feature extraction^a^.

Model	Parameter count (M)	FLOPS^b^ (giga)	Validation accuracy (%)	mAP^c^	*F*_1_-score	Training time, minutes	Inference time, seconds
AlexNet	61.1	*0.71* ^d^	80.5 (79.4-81.6)	82.8 (81.7-83.9)	81.4 (80.3-82.5)	151	39
VGG16	138.36	15.47	83.4 (82.3-84.5)	86.6 (85.6-87.6)	84.5 (83.5-85.5)	258	47
VGG19	143.67	19.63	86.1 (85.1-87.1)	86.1 (85.1-87.1)	85.4 (84.4-86.4)	278	45
ResNet50	25.56	4.13	83.3 (82.2-84.4)	87.2 (86.2-88.2)	84.6 (83.6-85.6)	258	59
InceptionV4	42.62	6.12	50.3 (48.9-51.7)	76.7 (75.5-77.9)	66.4 (65.0-67.8)	310	99
DinoV2-Base	85.51	21.96	73.3 (72.0-74.6)	76.3 (75.1-77.5)	73.8 (72.5-75.1)	502	60
Swin-Base	87.7	15.17	86.1 (85.1-87.1)	86.8 (85.8-87.8)	86.4 (85.4-87.4)	494	112
DinoV2-Small	21.52	5.52	70.4 (69.1-71.7)	73.9 (72.6-75.2)	71.1 (69.8-72.4)	182	59
Swin-Tiny	28.27	4.37	60.3 (58.9-61.7)	76.7 (75.5-77.9)	58.8 (57.4-60.2)	175	35
GoogLeNet	*6.8* ^d^	1.51	*95.9* (*95.3-96.5*)^d^	*89.2* (*88.3-90.1*)^d^	*95.1* (*94.5-95.7*)^d^	*57* ^d^	*22* ^d^

a Performance metrics are reported as mean (95% CI).

b FLOPs: Floating Point Operations.

c mAP: mean average precision.

d The values in italics indicate the best performance or efficiency in the comparative results.

### Performance Evaluation for Feature Extraction Model

The feature extraction model underwent training for 150,000 iterations. To evaluate its performance, feature extraction was first applied to all images in both the training and test datasets. Next, the feature vectors from the test dataset were individually compared and ranked against those from the training dataset to measure the similarity between the respective images. Notably, an image was considered relevant (with rel(*k*)=1 in AP) if its category matched that of the query image.

A summary of the evaluation metrics, including mAP and Precision@10, is shown in Table 3 for each image class. These metrics assess the performance and retrieval quality of the model for each image class as well as overall. The overall mAP for the study was 89.16%, with a Precision@10 of 94.08%. The best performance was observed in the normal brain category, achieving a mAP of 94.95% (95% CI 94.32‐95.58) and a Precision@10 of 97.57% (95% CI 97.13‐98.01), indicating high reliability in identifying normal tissues.

The results show that the overall classification performance of this model was excellent, but its mAP for CBMIR was only 89.16%. This disparity highlights the difference between classification and image retrieval in practical terms. In classification, the main training objective of the AI model is to identify the tumor class to which the current image belongs. However, in image retrieval, the training objective must consider the similarity (distance relationship) between the current image and other images to learn the relationships between the features of each image in the pair of images. These are 2 conceptually distinct tasks.

**Table 3.** Evaluation results by tumor class^a^.

Tumor class	mAP^b^ (%)	Precision@10 (%)
Metastasis	88.01 (87.08-88.94)	92.47 (91.71-93.23)
Glioma	88.33 (87.41-89.25)	92.70 (91.95-93.45)
Meningioma	90.94 (90.12-91.76)	96.20 (95.65-96.75)
Pituitary adenoma	87.76 (86.82-88.70)	98.01 (97.61-98.41)
Vestibular schwannoma	87.89 (86.95-88.83)	94.51 (93.86-95.16)
No tumor contour	87.39 (86.44-88.34)	89.31 (88.42-90.20)
Normal brain	94.95 (94.32-95.58)	97.57 (97.13-98.01)
Overall	89.16 (88.27-90.05)	94.08 (93.40-94.76)

a Performance metrics are reported as mean (95% CI).

b mAP: mean average precision.

### Performance Evaluation for Tumor Classification

**Figure 3.**
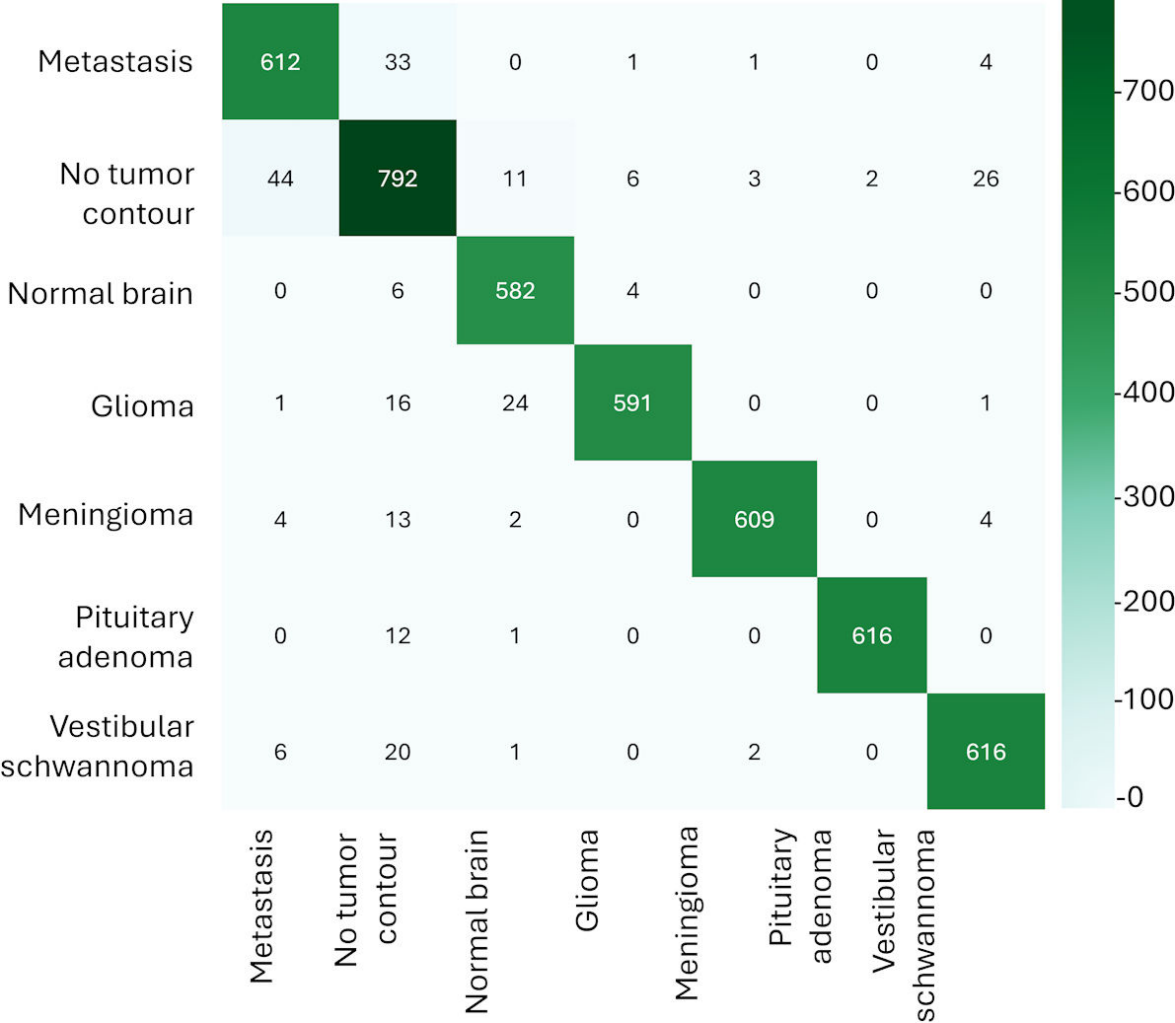
Confusion matrix for classes of brain tumors.

The main purpose of this study was to use an AI model to extract feature vectors for image retrieval. To realize this purpose, we chose to extend the ArcFace loss function, which is based on the softmax loss function, using a margin of 38 and a scale of 64, as the classification for a model that can theoretically be used for image classification. Therefore, this study also investigated the performance of the AI model for classification tasks. Table 4 shows the precision, recall, and *F*_1_-score for each class, while Figure 3 provides the confusion matrix for each tumor class.

**Table 4.** Classification performance evaluation for each tumor class.

Tumor class	Precision, %	Recall, %	*F*_1_-score, %
Metastasis	91.75	94.01	92.87
Glioma	98.17	93.36	95.71
Meningioma	99.02	96.36	97.67
Pituitary adenoma	99.68	97.93	98.80
Vestibular schwannoma	94.62	95.50	95.06
No tumor contour	88.79	89.59	89.19
Normal brain	93.72	98.31	95.96
Overall	95.11	95.01	95.04

### Performance Comparison at Various Embedding Dimensions

In our study, ArcFace loss was used not merely for classification but as a feature embedding mechanism to ensure that images belonging to the same tumor class are mapped closer together in the embedding space, while those from different classes are more widely separated. This results in well-clustered and discriminative feature vectors, which form the necessary basis for our similarity-based retrieval. Hence, although these are distinct tasks, they are synergistically integrated within the same CBMIR system: classification helps structure the embedding space, thereby significantly enhancing the discriminative power and ultimately the retrieval performance of the system.

 We initially drew inspiration from a study [37] in which the optimal embedding dimension for their specific objective of facial recognition was determined to be 128. However, we recognize that this dimension may not necessarily be optimal for brain tumor classification. Consequently, we undertook a comprehensive comparison of various embedding dimensions, as outlined in Table 5. The findings of this analysis unequivocally indicated that 256 dimensions yielded the highest performance for our retrieval task.

Furthermore, we note that the disparities in performance across dimensions, ranging from 64 to 512, were relatively marginal. This observation is particularly significant when considering the cost-effectiveness of deploying the model on devices with limited computational capabilities. Opting for a dimensionality of 64 can substantially curtail computational overheads while still maintaining respectable performance levels.

**Table 5.** Performance comparison of different embedding dimensions.

Embedding size	Overall mAP, %
64	88.16
128	88.76
256	89.16^a^
512	89.14

a Indicates the best performance in different various embedding dimensions.

### Feature Vector Projection

**Figure 4.**
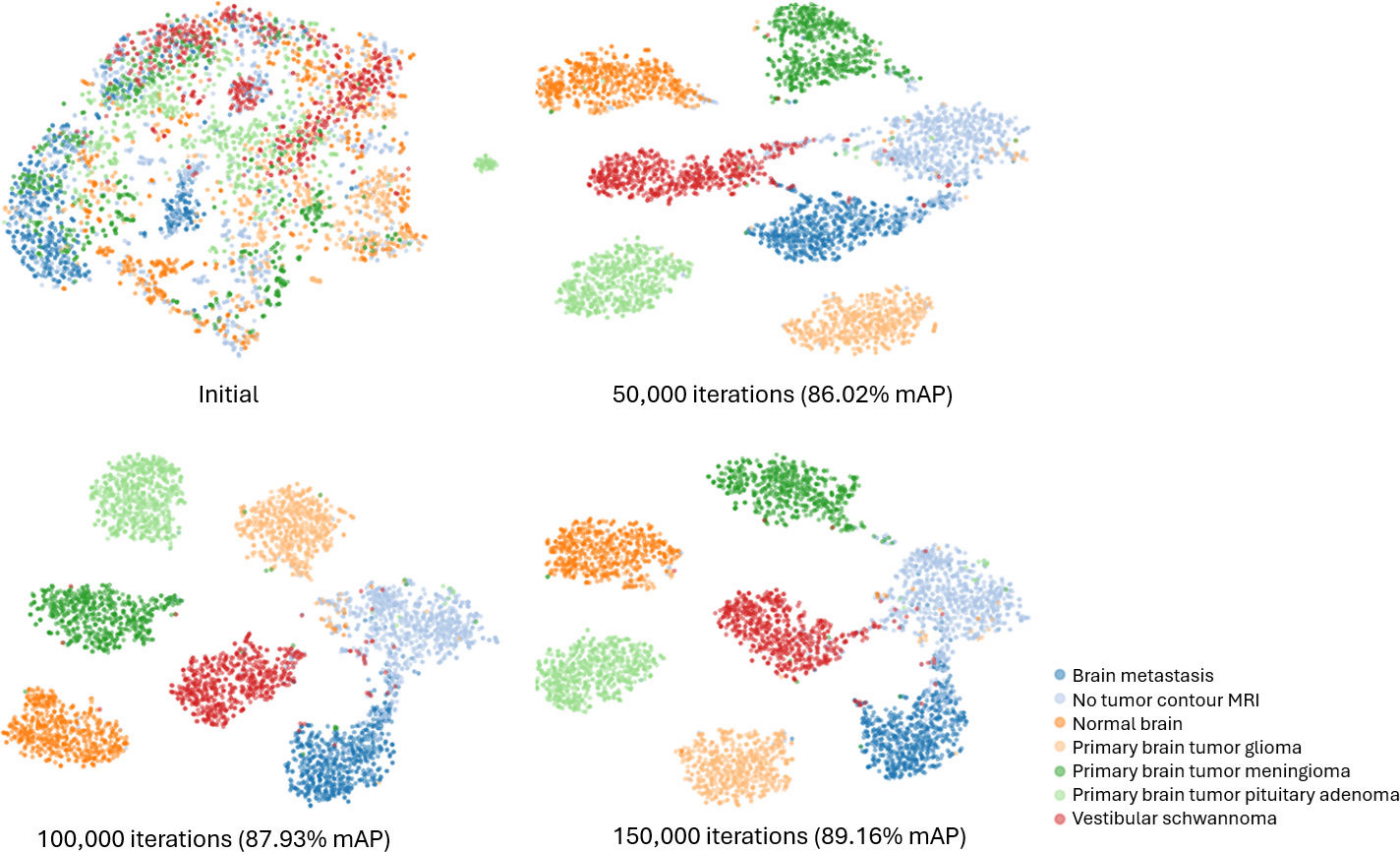
Visual representation of feature vector projection using t-distributed Stochastic Neighbor Embedding across various training iterations. The projection was implemented with 3 components, a perplexity of 30, 1000 iterations, and the Euclidean metric. This visualization is intended to illustrate trends in feature learning rather than to provide quantitative evidence of class separation. mAP, mean average precision; MRI, magnetic resonance imaging.

Our methodology consolidated similar feature vectors while actively distancing dissimilar ones. While mAP serves as a useful indirect metric for assessing effectiveness, the feature vector coherence can be qualitatively assessed through projection. To qualitatively visualize the evolution of feature representations during training, we used t-distributed Stochastic Neighbor Embedding to project high-dimensional feature vectors into a lower-dimensional space. As a stochastic dimensionality reduction method, t-distributed Stochastic Neighbor Embedding is sensitive to parameter settings and is used here solely for qualitative inspection of learning trends rather than as quantitative evidence of feature space cohesion or class separability. The visual representation of feature vector projection is illustrated in Figure 4.

Figure 4 illustrates that the initially untrained feature vectors, denoted as “Initial,” exhibit a scattered pattern with minimal cohesion among different classes. However, as training progresses through 50,000, 100,000, and 150,000 iterations, we observe a gradual convergence of feature vectors within each class, reflecting increasing cohesion. Concurrently, an observable qualitative visual separation implies that feature vectors from different classes become more distantly grouped, highlighting the model’s progressive learning and adaptability.

### System Integration With PACS

Our system operates as follows. Users can initiate image queries from the PACS and subsequently retrieve and display these images within BlueLight via the QIDO (query based on ID for DICOM Objects) and WADO (web access to DICOM objects) protocols, respectively. To use the CBMIR functionality, users provide the StudyInstanceUID, SeriesInstanceUID, and SOPInstanceUID for the specific DICOM image they require.

**Figure 5.**
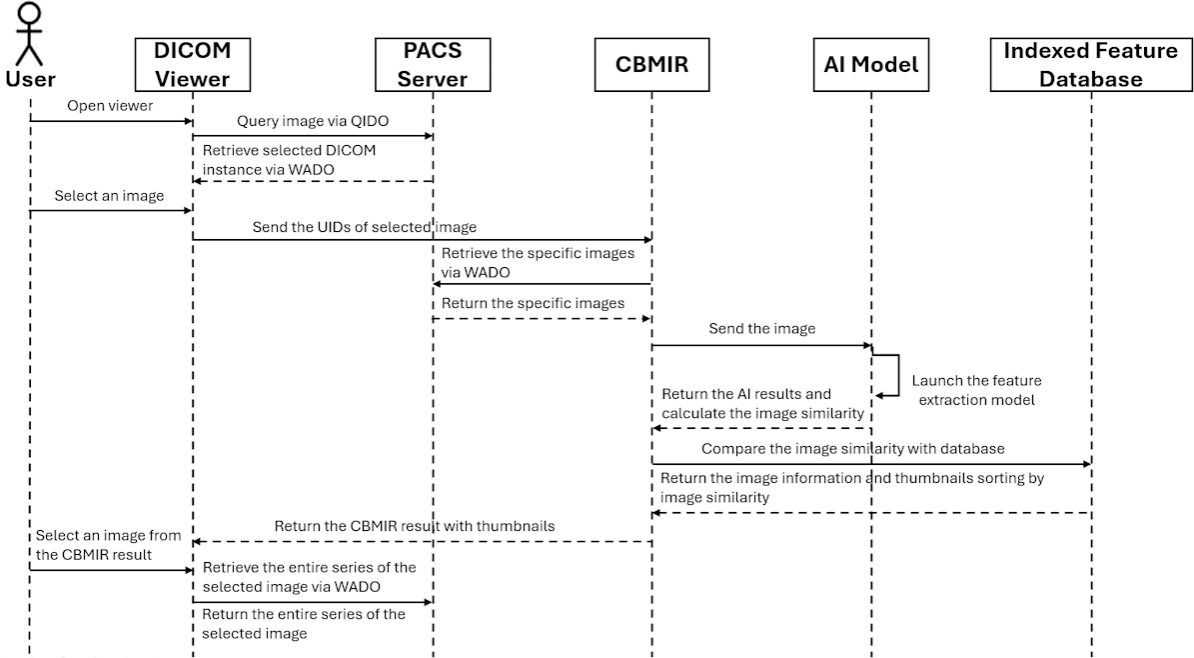
Sequence diagram for the integration of content-based medical image retrieval with a picture archiving and communication system. AI: artificial intelligence; CBMIR: content-based medical image retrieval; DICOM: Digital Imaging and Communications in Medicine; PACS: picture archiving and communication system; UIDs: unique identifiers.

Once these details are specified, the CBMIR system retrieves the corresponding DICOM image from the PACS using WADO and then processes this image to calculate the CBMIR results, returning the top 10 images that bear the highest similarity to the input image. The operational process and data workflow are depicted in Figure 5.

**Figure 6.**
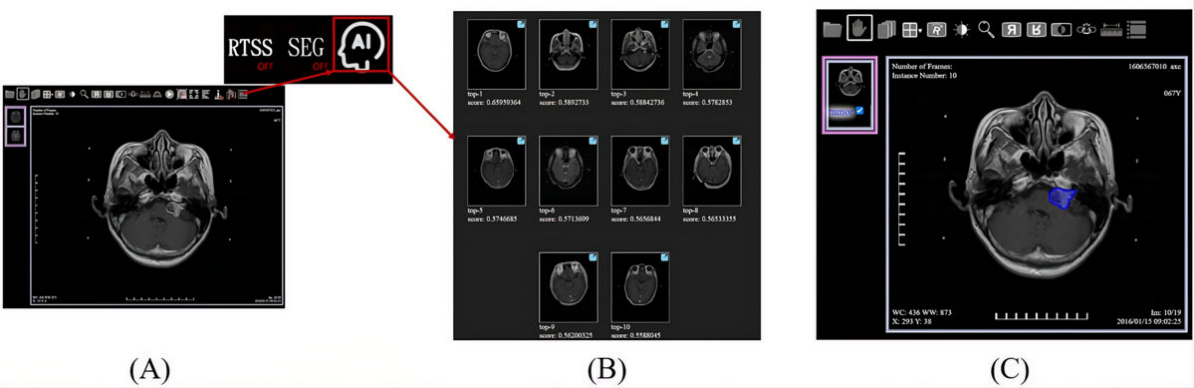
The user interface of the content-based medical image retrieval (CBMIR) services is integrated into a web Digital Imaging and Communications in Medicine viewer. (A) Before performing the CBMIR system; (B) Top 10 retrieval results ordered by descending similarity scores. A higher score denotes greater visual similarity between the query and the retrieved images; (C) Retrieve the selected whole series of images from picture archiving and communication system.

A demonstration video is provided in Multimedia Appendix 1, and a screenshot of the user interface of the CBMIR client prior to executing a search is shown in Figure 6A. To enhance the successful integration of our CBMIR system into clinical workflows, we integrated it with an open-source DICOM viewer known as BlueLight [34] and the DICOM server Raccoon [38]. This client application communicated with a PACS-supporting DICOMweb, significantly simplifying user access to and interpretation of brain tumor images, including the display of top retrieval results ordered by similarity scores (Figure 6B). Moreover, considering that brain images are inherently 3D, a single image may not always convey the complete information for a whole series of images. The system’s workflow ensures that the entire series of images associated with a case is retrieved from the PACS, rather than just a single instance (Figure 6C).

### Performance of CBMIR System

During the usage of the CBMIR system, the “time” and “stability” of querying for similar images can significantly impact the overall operational process. Therefore, this study conducted a performance evaluation of the CBMIR system. The response time represents the end-to-end round trip for image retrieval, including WADO communication, database execution, and network transfer latency. The overall response time evaluation involved the following steps: (1) sending image to the CBMIR system, (2) retrieving images from PACS, (3) extracting image feature vectors using the CBMIR AI model, (4) indexing image feature vectors into the database, (5) querying for similar images within the database, and (6) assessing the response time back to the user.

**Figure 7.**
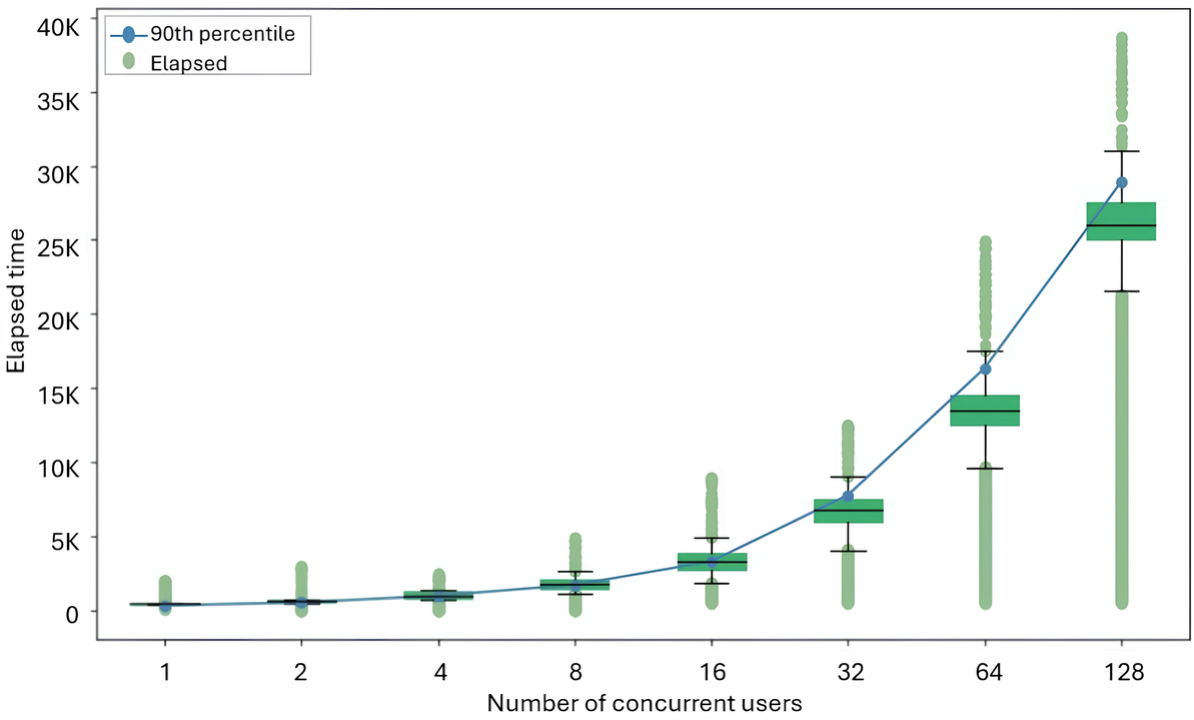
Relationship between the elapsed response time and the number of concurrent users in the content-based medical image retrieval system evaluation. The green box-and-whisker plots and scatter points display the statistical distribution of the response time (median, quartiles, and extreme values), while the blue line graph indicates the 90th percentile response time.

The stability assessment involved simulations with 2^*n* users, ranging up to 256 concurrent users, using the CBMIR system. The evaluation results are shown in Table 6; a box-and-whisker plot depicting the evaluation results is shown in Figure 7.

Additionally, as no system crashes or errors were observed for user counts ranging from 1 to 128, this study conducted further testing, revealing that when more than 256 users simultaneously used the system, all requests began to experience 30-second time-outs, the default value of request time-out for all web browsers, thereby all requests failed. The results demonstrate that as the number of concurrent users increases, the response time exhibits a nonlinear growth. Specifically, under extreme stress testing conditions (exceeding 64 simultaneous users), the variability in response time expands significantly. When the concurrent load surpasses 256 users, the system reaches its current architectural capacity limit, resulting in request time-outs (exceeding 30 seconds). These findings delineate the operational boundaries of the current implementation, indicating that while the system is stable for routine, low-concurrency clinical usage, high-concurrency scenarios impose a significant load that impacts real-time stability.

**Table 6.** Response time results under various user loads.

Users	Response time, ms		
	Average	Maximum	90th percentile
1	273.98	1373	333.00
2	434.48	2696	531.00
4	918.89	2233	1187.00
8	1758.38	4423	2219.00
16	3225.49	8230	3903.10
32	6432.68	12,541	7775.40
64	13629.18	24,391	16337.40
128	25672.47	39,196	28909.10

## Discussion

### Principal Findings

We developed and evaluated a CBMIR system tailored for brain tumor detection within a PACS environment. The results demonstrate a substantial improvement in retrieval accuracy enabled by the innovative deep learning architecture, enhancing feature discriminability. By integrating our AI model with 2 open-source tools—Raccoon for backend DICOM management and BlueLight for frontend web-based image viewing—our CBMIR system provides seamless, real-time decision support directly within the clinician's workflow. This practical integration helps bridge the gap between advanced AI retrieval techniques and routine clinical practice. This achievement represents an important step toward establishing the CBMIR system as a valuable tool for both clinical diagnosis support and medical research.

#### Rationale for GoogLeNet Selection

We selected GoogLeNet as the backbone feature extractor for our CBMIR system due to its demonstrated optimal balance between clinical use and overall performance.

1. *Performance versus computational cost*: As Table 2 illustrates, while ViT-based and deeper CNN architectures are state of the art in many domains, their performance metrics on our specific medical imaging task are significantly lower than those achieved by GoogLeNet. This discrepancy is likely influenced by the interaction between model complexity and the relatively homogeneous characteristics of the dataset. In contrast, GoogLeNet’s moderate depth and multiscale receptive fields appear to facilitate more stable embedding learning and improved generalization in similarity-based retrieval scenarios.
2. *Overwhelming computational advantage*: GoogLeNet demonstrates a massive advantage in efficiency. Its Floating Point Operations are nearly one-tenth that of Swin (1.51 G vs 15.17 G), and its parameter count is approximately one-tenth that of the ViT models (6.8 M vs ≈85 M). Furthermore, its training time is significantly shorter (57 minutes vs ≈500 minutes).
3. *Critical clinical deployment consideration*: Given that a CBMIR system must be lightweight and support low-latency retrieval for real-time clinical use, efficiency is paramount. GoogLeNet provides a high mAP of 89.16% while maintaining minimal computational demands. Therefore, GoogLeNet was chosen as it achieves the best balance among accuracy, model compactness, and deployment efficiency, making it the superior foundation for our CBMIR architecture.

#### Heatmaps of Image Similarity

To provide interpretability for our CBMIR model’s retrieval decisions, we used a visualization technique based on Gradient-weighted Class Activation Mapping, adapted for deep similarity networks following the approach introduced by Stylianou et al [39].

**Figure 8.**
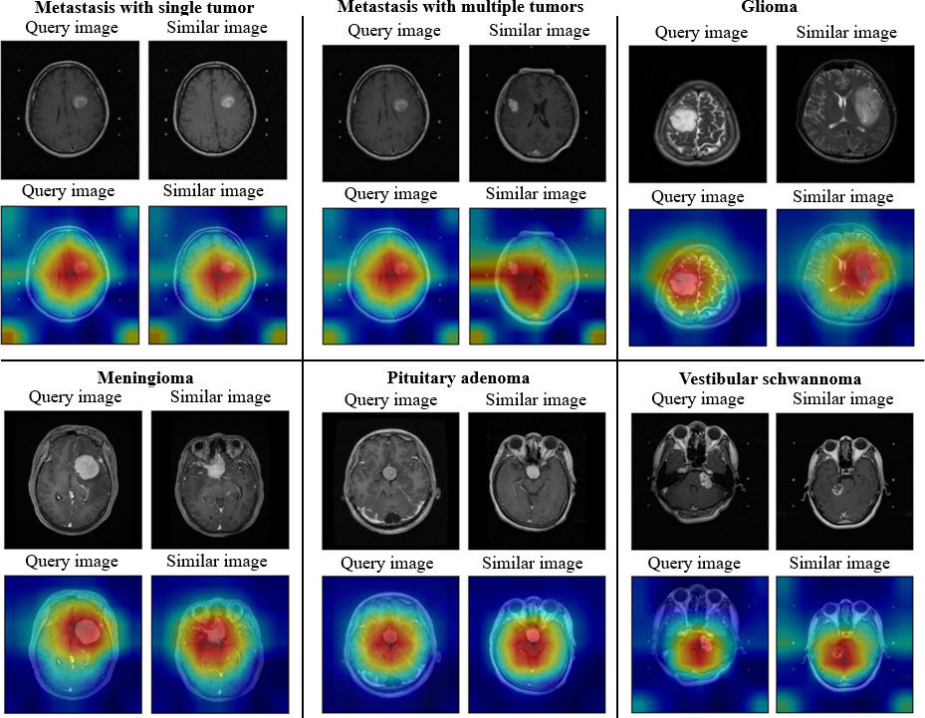
Heatmaps of similar images for various tumor classes: query image and retrieved similar image with corresponding heatmaps.

Our heatmaps aim to quantify the degree of feature matching between 2 images. Our methodology integrates Gradient-weighted Class Activation Mapping with the calculation of distances between the final convolutional layer and the pooling layer to gauge the level of attention or similarity across different regions within the images. In the visualization, regions exhibiting high similarity are represented in red, while those with lower similarity are depicted in blue. Figure 8 shows 5 examples of brain tumors, illustrating our primary goal of identifying images of brain tumors that share similarities. The ensuing sections showcase detailed heatmaps for each of the tumor classes.

For all tumor classes presented in Figure 8, except for the case of multiple tumors in metastasis, the examples used contain only a single tumor within the image. This choice was deliberate to ensure that the heatmaps clearly illustrate that retrieval similarity is based on matching the pathological features of the tumor (where the hot spot is located), rather than simply reflecting class probabilities as in a classification task. The high feature consistency observed between the query and retrieved images across these single-lesion cases strongly supports this interpretation. However, it is essential to note that while this analysis explains the underlying mechanism, the study does not draw definitive clinical conclusions regarding these results. It should be emphasized that these heatmaps are presented for qualitative illustration only. Determining whether such activation patterns correspond to meaningful anatomical features or to nonanatomical factors would require systematic image-by-image analysis, which is beyond the scope of this study.

#### Single Versus Multiple Tumors (Metastasis Cases)

We included metastasis cases with both single and multiple tumors within a single image. The heatmaps illustrate visually different activation patterns between these 2 scenarios. However, the primary focus of our analysis remains the observed differences in image similarity behavior between metastasis cases with multiple tumors and single-tumor cases, rather than the anatomical interpretation of activation maps.

1. *Single tumor*: In single-tumor cases, the query image heatmap shows a visually concentrated activation region (red or yellow) localized around the dominant lesion. The corresponding retrieved image exhibits a similarly localized activation pattern. This observation reflects a spatially focused activation pattern in both the query and retrieved images.
2. *Multiple tumors*: In multiple-tumor cases, the activation patterns appear more spatially distributed. While high-activation regions remain present near individual lesions, the heatmaps often extend into broader areas of the image. The similarity image heatmap exhibits horizontally distributed activation patterns or band-like structures. These patterns indicate a more dispersed activation distribution in images containing multiple lesions.

#### Clinical Considerations for Cross-Class Image Similarity

Our heatmap analysis differs from traditional methods, which often focus only on areas of interest within a single image for classification. Our objective is to describe and quantify the similarity between 2 images in terms of mutual focus on specific regions, an understanding pivotal for elucidating the model’s retrieval effectiveness. Considering clinical evaluation, 2 radiologists (KHK and WYG) used the hospital’s DICOM viewer to cross-validate brain tumor cases and assess the correctness of similarity scores between different tumor classes.

We acknowledge that highly similar images from the query results may not always belong to the same tumor class. Indeed, in some instances, the similarity score may be substantially higher for images assigned to other tumor classes. These cases indicate a significant degree of visual and structural image similarity, even if the retrieved image’s classification label may not be totally accurate. The similarity score is calculated for the entire image. While a partial score calculated from the tumor lesion may result in a high score, it is worth noting that when 2 images share a similar location in an axial brain slice, the similarity of surrounding organs and structures may yield a higher final score than that of the tumor lesion alone.

This result shows a clinical potential use in identifying shared characteristics among images across tumor classes. However, it must be emphasized that definitive conclusions regarding these results were not drawn in this study. To mitigate this potential disparity, the user interface has been thoughtfully crafted to label these images accordingly, providing a clear notification to the user regarding this outcome.

#### Normal Brain Versus No Tumor Contour

From a clinical perspective, the “Normal Brain” class represents images from patients without a brain tumor diagnosis, whereas the “No Tumor Contour” class consists of image slices acquired from patients with known brain tumors in which no visible lesion is present on the specific slice. Although these 2 classes may appear visually similar at the slice level, they differ fundamentally at the case level, which explains the observed confusion in similarity-based retrieval. Specifically, while the “Normal Brain” class achieves a high mAP of 94.95%, the “No Tumor Contour” class yields a lower mAP of 87.39%, which is below the overall average of 89.16%.

This confusion reflects a realistic diagnostic scenario in routine clinical practice, where individual slices without visible lesions may be indistinguishable from normal brain images. This observation highlights the system’s ability to handle visually overlapping categories in routine clinical practice. The inclusion of these classes underscores the system’s ability to handle visual overlaps based on deep feature embeddings learned from pixel-level representations, rather than pathology-specific descriptors. Consequently, the reported mAP reflects the global coherence of the learned visual embedding space across diverse clinical categories.

It should be noted that this behavior does not imply a false-negative diagnostic result, as the proposed CBMIR system is designed for similarity-based image retrieval rather than automated diagnosis. Instead, it underscores the necessity of contextual interpretation at the case or series level when reviewing retrieved images. To mitigate potential clinical misunderstanding, the system retrieves the complete image series rather than a single slice, allowing clinicians to examine adjacent slices where lesions may be present. The interface further indicates that tumor annotations exist elsewhere within the retrieved series.

### Limitations

While this study successfully developed a high-performing CBMIR system and integrated it into PACS, there are limitations regarding dataset characteristics, system scalability, and model interpretability. First, regarding the dataset characteristics and partitioning, this study used a dataset spanning a long retrospective period (2000‐2017) from a single medical center, acquired primarily on 1.5T scanners. Although this provided consistency in clinical protocols and image quality, it may limit the model’s generalizability. In particular, robustness across different scanner manufacturers, field strengths (eg, 3T), and heterogeneous acquisition protocols remains to be systematically validated. Given the increasing prevalence of 3T scanning in modern neuro-oncology, the model’s performance on higher-resolution and advanced acquisition protocols warrants further investigation, especially in multicenter clinical settings.

To ensure balanced class distributions for benchmarking, we used a random split strategy. Unlike a prospective temporal split, this approach introduces a potential for “future leakage” and may not fully simulate clinical deployment timelines. A temporal split, in which earlier data are used for training and later data for testing, may better reflect real-world clinical deployment scenarios and provide a more stringent evaluation of generalization across time. However, such a design requires strict longitudinal harmonization of acquisition protocols, and scanner variations could provide a more stringent assessment of real-world generalization and will be a potential improvement in future studies.

Second, regarding system latency and scalability, although GoogLeNet was selected for its relatively low computational cost, the processes of calculating feature vectors and similarity computation create a computational bottleneck under heavy workloads. Our stress testing demonstrates that response time increases substantially under high concurrency, exceeding 13 seconds for 64 simultaneous users and approaching 26 seconds for 128 users. Such latency is incompatible with real-time clinical workflows and therefore represents a critical limitation of the current implementation.

Third, regarding the analysis of metastasis cases with multiple tumors, we observed distinct horizontal bands of high activation in the heatmaps. While these patterns may suggest distributed feature attention, we cannot definitively rule out the possibility that they represent acquisition or reconstruction artifacts (eg, motion or noise). Consequently, this ambiguity serves as a limitation regarding the model’s focus and explainability in complex, multilesion scenarios.

Fourth, to accommodate the input requirements of the pretrained GoogLeNet architecture, the original 512×512 pixels images were resized to 224×224 pixels before feature extraction. This downsampling inevitably reduces spatial resolution and may obscure fine-grained texture details that could be relevant for differentiating certain tumor subtypes. While the loss of fine-grained texture information may affect the representation of subtle local patterns, the system is designed to capture global and midlevel feature similarities that are sufficient for retrieval tasks within the intended clinical workflow. Nonetheless, this resolution reduction is a limitation of the current implementation.

Fifth, similarity in our CBMIR system does not rely on predefined morphological or pathological criteria (eg, tumor size, location, and texture). Instead, it is quantified by computing the Euclidean distance between high-dimensional deep embeddings derived directly from pixel-level representations. Consequently, similarity ranking is fundamentally driven by pixel-derived feature distances, rather than by pathological labels or manually defined structural attributes.

Finally, the retrieval performance metrics reported in this study were computed using Euclidean distance across the full test set to provide standardized and reproducible benchmarking. In contrast, the deployed CBMIR system uses an approximate nearest neighbor search strategy based on HNSW graphs to ensure low-latency, real-time responses and system scalability. The use of HNSW enables efficient similarity search for unseen queries without requiring their prior inclusion in a precalculated distance matrix. While this approximate nature may lead to minor deviations in retrieval precision or recall compared with an exhaustive search, such a trade-off is essential for practical clinical usability, effectively balancing theoretical accuracy with the demands of real-time medical decision support.

### Future Work

Building upon the current findings and addressing the identified limitations, we propose the following three main directions for future research:

1. *Expansion to multicenter dataset validation*: Future work will prioritize incorporating image data from diverse health care institutions to test and fine-tune the model’s robustness. Training and validating the system on multicenter datasets will significantly enhance the model’s generalizability and practical usefulness.
2. *Exploration of modern AI model*: Although preliminary experiments showed modern AI models, for example, Swin and DinoV2, to be less computationally efficient for our specific task, their inherent strength in capturing long-range dependencies warrants further investigation. Future research may explore methods to optimize or prune these AI models to harness their superior feature learning capabilities without unduly sacrificing computational efficiency.
3. *Integration of multimodal retrieval*: The current system relies solely on visual features. In clinical practice, structured and unstructured radiology reports provide critical contextual information. Future work may explore multimodal deep learning models to combine image features with textual features, enabling more comprehensive and accurate multimodal retrieval functionality.

### Conclusions

This study represents the design, implementation, and evaluation of a CBMIR tailored for the classification and retrieval of brain MR images. We successfully developed a CBMIR system focusing on structured tumor classification and incorporating deep learning–based feature extraction, including GoogLeNet combined with ArcFace Loss to enhance the discrimination of image similarities and differences, thereby improving retrieval relevance. In addition, GeM pooling was used as the feature aggregation strategy and evaluated in comparison with conventional average pooling and maximum pooling methods. These results indicate that GeM pooling provides a more refined representative embedding of image features, contributing to improved retrieval accuracy and robustness in the CBMIR system. To facilitate practical deployment, the training workflow was designed as a streamlined, single-stage process, reducing complexity and enabling efficient model preparation. The system was trained using a dataset that includes both tumor-positive and tumor-negative brain MR images, reflecting the diversity of cases encountered in routine clinical practice. Finally, the proposed CBMIR system was successfully integrated with the open-source web DICOM viewer BlueLight with a PACS. The retrieved results were able to be communicated with a PACS to retrieve corresponding medical images for display in a web DICOM viewer, demonstrating the feasibility of integrating CBMIR functionality into existing clinical imaging workflows.

## Supplementary material

10.2196/78300 Multimedia Appendix 1 Demonstration of the content-based medical image retrieval (CBMIR) system integration. This video illustrates the complete workflow of the CBMIR system integrated with the BlueLight DICOM viewer and picture archiving and communication system. The user interface shown in this demonstration displays the raw Euclidean distance as the “score” (where lower values indicate higher similarity).

## Abbreviations

AI: artificial intelligence
AP: average precision
BN: BatchNorm
CBMIR: content-based medical image retrieval
CNN: Convolutional Neural Network
DICOM: Digital Imaging and Communications in Medicine
GeM: generalized mean
HNSW: Hierarchical Navigable Small World
mA: mean average precision
MR: magnetic resonance
PACS: picture archiving and communication system
PReLU: parametric rectified linear unit
QIDO: query based on ID for DICOM Objects
TL: transfer learning
TVGH: Taipei Veterans General Hospital
VGG19: Visual Geometry Group model 19
ViT: Vision Transformer
WADO: web access to DICOM objects
